# Regulation of the AbrA1/A2 Two-Component System in *Streptomyces coelicolor* and the Potential of Its Deletion Strain as a Heterologous Host for Antibiotic Production

**DOI:** 10.1371/journal.pone.0109844

**Published:** 2014-10-10

**Authors:** Sergio Rico, Ana Yepes, Héctor Rodríguez, Jorge Santamaría, Sergio Antoraz, Eva M. Krause, Margarita Díaz, Ramón I. Santamaría

**Affiliations:** 1 Instituto de Biología Funcional y Genómica/Departamento de Microbiología y Genética, Consejo Superior de Investigaciones Científicas/Universidad de Salamanca, Salamanca, Spain; 2 Institute for Molecular Infection Biology, Julius-Maximilians-Universität Würzburg, Würzburg, Germany; 3 Universitätsbibliothek Marburg, Marburg, Germany; University of Strathclyde, United Kingdom

## Abstract

The Two-Component System (TCS) AbrA1/A2 from *Streptomyces coelicolor* M145 is a negative regulator of antibiotic production and morphological differentiation. In this work we show that it is able to auto-regulate its expression, exerting a positive induction of its own operon promoter, and that its activation is dependent on the presence of iron. The overexpression of the *abrA2* response regulator (RR) gene in the mutant Δ*abrA1/A2* results in a toxic phenotype. The reason is an excess of phosphorylated AbrA2, as shown by phosphoablative and phosphomimetic AbrA2 mutants. Therefore, non-cognate histidine kinases (HKs) or small phospho-donors may be responsible for AbrA2 phosphorylation *in vivo*. The results suggest that in the parent strain *S. coelicolor* M145 the correct amount of phosphorylated AbrA2 is adjusted through the phosphorylation-dephosphorylation activity rate of the HK AbrA1. Furthermore, the ABC transporter system, which is part of the four-gene operon comprising AbrA1/A2, is necessary to de-repress antibiotic production in the TCS null mutant. Finally, in order to test the possible biotechnological applications of the Δ*abrA1/A2* strain, we demonstrate that the production of the antitumoral antibiotic oviedomycin is duplicated in this strain as compared with the production obtained in the wild type, showing that this strain is a good host for heterologous antibiotic production. Thus, this genetically modified strain could be interesting for the biotechnology industry.

## Introduction

The regulatory systems involved in secondary metabolite production in *Streptomyces* are key targets for metabolic engineering possibilities [1]. The signals detected by pleiotropic regulators, which control both morphological differentiation and antibiotic production in this genus, are ultimately transmitted to the pathway-specific regulators that eventually switch on/off the transcription of the biosynthesis-related genes. A close relationship between the morphogenetic program and antibiotic production has been described in this genus [2]. TCSs are widespread signaling regulators that mediate cascades with pleiotropic effects. The existence of many TCSs in *Streptomyces* correlates with many alternative pathways to respond to all types of physiological situations and environmental changes [3].

In all canonical TCSs, there must be a signal sensed by the histidine kinase (HK), which then becomes auto-phosphorylated in the histidine residue. It is not easy to determine the signal that a specific TCS responds to. To date only a few TCS-activating signals have been described in *Streptomyces* and hence understanding the control of the regulatory networks remains a challenge [3], [4].

Phosphorylated HK has the ability to phosphotransfer phosphate to an aspartic residue of the response regulator (RR), eliciting a conformational change and its activation. Many studies addressing the partner fidelity of this process have been conducted. In theory, the kinetics are more favorable between partners, although in some cases it is possible that an HK might phosphorylate different RRs [5]. In fact, a bioinformatic tool has been developed to predict inter-species crosstalk [6]. The phosphorylation of the RRs may also take place in the absence of its cognate HK by small phospho-donors such as acetylphosphate, carbamoylphosphate and phosphoramidate [7], [8]. The half-life of the phosphorylated RRs may vary from seconds to hours, and dephosphorylation is carried out by phosphatases. Sometimes, external proteins carry out this reaction, yet in many cases the cognate HK is responsible and it also exhibits phosphatase activity against the corresponding RR. In any case, the phosphorylation state of the RR is crucial for its activity and must be strictly regulated. The amount of RR in the cells is also controlled at transcriptional level. Remarkably, a positive feedback is often found in the expression of the RR, where the active form of the regulator activates its own expression [9].

In *Streptomyces coelicolor* M145, AbrA1/A2 TCS acts as a pleiotropic negative regulator of antibiotic production and differentiation [10]. The absence of both genes, *abrA1* and *abrA2*, enhances actinorhodin (ACT), undecylprodiginine (RED) and calcium-dependent antibiotic (CDA) production, and it also accelerates the developmental program. The *abrA1* and *abrA2* genes of this TCS are part of a four-gene operon harboring another two upstream genes encoding an ABC transport protein system (*SCOs1742/1743*).

In this work we further characterize the AbrA TCS. First, we were interested in exploring which signal was able to trigger the AbrA pathway by activating the HK AbrA1 and we observed that this system depends on Fe. Second, we demonstrate that AbrA system exerts a positive auto-regulation and that a basal expression of the adjacent ABC system is necessary for the de-repression observed in the mutant lacking the TCS genes of the operon. Additionally, our results support the notion that AbrA1 might control the phosphorylation state of AbrA2 through both phosphorylation and dephosphorylation activities. Some putative cross-talk can also be proposed in view of the intermediate phenotypes observed in the individual mutants Δ*abrA1* and Δ*abrA2*, and hence non-cognate HKs and RRs might act when the corresponding partners are absent, although with less efficiency.

Finally, we show the potential of using the Δ*abrA1/A2* strain to express secondary metabolites heterologously, offering a new tool for the biotechnological industry. This strain is able to over-produce the antitumoral oviedomycin of *S. antibioticus* [11], [12], [13] in an active configuration.

## Materials and Methods

### Strains, media and culture conditions

*Escherichia coli* strains BL21(DE3) [14], BW25113/pIJ790 (containing the λRed system) [15], and non-methylating ET12567/pUZ8002 (containing the *tra* genes) [16] were grown in Luria–Bertani (LB) liquid broth or on LB agar. The *S. coelicolor* M145 and mutant strains were grown on NA, R2YE, MSA, PGA, YEPD and NMMP [17]. Several modifications of the NMMP composition were used (complete medium: 5 g/L glucose, 2 g/L (NH_4_)SO_4_, 0.6 g/L MgSO_4_, 5 g/L casamino acids, 15 mL/L 0.1 M NaH_2_PO/Na_2_HPO buffer, pH 6,8, 1 mL/L trace element solution (1 g/L ZnSO_4_.7H_2_O, 1 g/L FeSO_4_.7H_2_O, 1 g/L MnCl_2_.4H_2_O, 1 g/L CaCl_2_), 20 g/L agar) [18]. Liquid cultures were performed in 100-mL three-baffled flasks with 15 mL of medium each. When necessary, the medium was supplemented with antibiotics (100 µg mL^−1^ of ampicillin, 50 µg mL^−1^ of apramycin, 50 µg mL^−1^ of kanamycin, 10 µg mL^−1^ of thiostrepton, 20 µg mL^−1^ of hygromycin, 20 µg mL^−1^ of neomycin, 25 µg mL^−1^ of chloramphenicol, and 25 µg mL^−1^ of nalidixic acid).

### Mutant constructions (TCS knockouts)

The PCR-targeting system established by Gust *et al.* [19] was used to replace the corresponding gene or genes by an apramycin (*aac(3)IV* gene) resistance cassette, later eliminating the resistance cassette using Flp recombinase. Mutagenic cassettes were amplified using the specific primers for each gene (Table S1) and the pIJ773 plasmid as template. The SRG-059/SRG-045 or SRG-046/SRG-058 primer pairs were used for the mutagenic cassettes to replace *SCO1744* or *SCO1745* respectively. To delete *SCOs1742/43*, the oligonucleotides used were AY-043/AY-044 in a first PCR, and AY-045/AY-046 in a second PCR to extend the flanked regions of the ORFs to be deleted. To construct the mutagenic cassette for *SCOs1742-45*, the same forward primer (AY-043) as that employed for the disruption of *SCO1742/43* was used, and as the reverse primer AY-006P was used both for the first and for the second PCR. The resistance gene replaced the corresponding ORFs of the cosmid SCI11 with the corresponding mutagenesis cassettes in *E.coli* BW25223/pIJ90, and the mutant cosmids (ΔSCD11-2, ΔSCD11-3, ΔSCD11-4, and ΔSCD11-5) were transferred into *S. coelicolor* by intergeneric conjugation.

The different *Streptomyces* null mutants were isolated and confirmed by PCR and Southern assays.

### Plasmid constructions

The plasmids and cosmids used are listed in Table S2. The response regulator gene *abrA2* was amplified in a PCR reaction using primers AY-047 (including a NdeI site) and AY-048 (including a XhoI site) and cloned into NdeI/XhoI sites from the pXHis1 plasmid [20], generating an intermediate plasmid designated pXabrA2His. The BglII fragment containing the *abrA2* gene (Hisx6-tagged) under the *xysAp* promoter [21] was cloned into the same site from the *Streptomyces* pIJ702 plasmid [22], yielding pTXAbrA2.

To obtain plasmids pTXabrA2-DA and pTXabrA2-DE, D55A or D55E point mutations were introduced using pairs of oligonucleotides specific for each mutation in a first step of an overlapping PCR: AY-047/AY-104 (5′ gene fragment DA) and AY-103/AY-048 (3′ gene fragment DA) or AY-047/SRG051 (5′ gene fragment DE) and AY-048/SRG-050 (3′ gene fragment DE). Then, using these two overlapping fragments for D55A or D55E as a template, a second PCR was carried out with AY-047/AY-048 and the final mutated genes were introduced by NdeI/XhoI digestion, replacing the non-modified version carried in pTXabrA2. In pTXabrA2-DADE, the mutant gene (D10A/D55E) was amplified using SRG-060/AY-048 oligonucleotides and the pTXabrA2-DE plasmid as template. This double mutant gene fragment was substituted as above in pTXabrA2.

For complementation studies, the integrative plasmids pHabrA1 and pHabrA2 were obtained by PCR amplification of *abrA1* using AY-035/SAM-001 oligonucleotides or *abrA2* (AY-047/AY-036) and replacing the *abrA1/A2* genes either by *abrA1* or by *abrA2*, respectively, in the pHabrA plasmid [10] with NdeI/XhoI digestion.

The new plasmids were introduced into the corresponding *Streptomyces* strains by protoplast transformation, as previously described [17].

The NdeI/XhoI fragment from pXabrA2His was introduced into pET22b to yield pETabrA2 for protein production and purification in *E. coli.* To purify the truncated AbrA2_N_ protein (141 aas) lacking the C-terminal fraction, the DNA encoding the N-terminal fraction was amplified using oligonucleotides AY-047 and AY-102 and cloned into the NdeI/XhoI sites of pET22b (pETabrA2_N_).

To obtain the pNA4 plasmid, the *abrA* operon promotor was amplified by PCR with AY-033 and AY-034, and cloned into the EcoRI/NdeI sites of pNX4, replacing the xylanase promotor.

### Nucleic acid manipulations

Plasmid isolation, restriction enzyme digestion, ligation, and transformation of *E. coli* and *S. coelicolor* were carried out using the methods of Sambrook *et al.* [23] and Kieser *et al.* [17], respectively. Total genomic DNA from *S. coelicolor* (gDNA) was isolated from 24–36 h cultures in TSB medium following the procedure described in Hopwood *et al.* [18], but scaled to 1–2 grams of mycelium.

For RNA extraction from the different strains, 160 mL of NMMP medium were inoculated in 500-mL baffled flasks with 4×10^6^ spores/mL and incubated at 30°C for 72 hours. Prior to RNA isolation, 20 mL of culture was harvested and suspended in RNA-protect Bacteria Reagent (Qiagen). Following mycelium lysis with lysozyme (15 mg/mL in TE at room temperature), three volumes of RLT buffer from the RNeasy mini plus kit (Qiagen) were added and mycelia were disrupted using Fast-prep (2 pulses of 10 seconds at maximum intensity, ice for 5 min between cycles). The lysate was clarified by centrifugation and the RNeasy mini Plus (Qiagen) kit was used to purify the RNA, following the manufacturer's specifications. The quality and concentration of RNA were assayed using spectrophotometric assays (Nanodrop ND1000).

RT-PCR was performed using 0.25 µg of RNA and iScript Reverse Transcription Supermix to obtain cDNA, following the manufacturer's specifications. The specific oligonucleotides AY-061 and AY-062 were used to analyze *SCO1742* expression in the different mutant strains for 35 cycles.

### Phosphorylation by low-molecular weight phosphor-donors

Phosphorylation reactions were carried out with 2.5 µg of AbrA2, AbrA2_N_, or DrrB_N_ protein, used as a control in the experiments [24]. The phosphorylation buffer was 50 mM Tris-HCl, pH 7.5, 100 mM NaCl, 10 mM MgCl_2_ and the low-weight phosphor-donors used were 20 mM acetylphosphate (Sigma), 100 mM carbamoylphosphate (Sigma) and 150 mM phosphoramidate (obtained by chemical synthesis as in Sheridan *et al*. [25]). The reactions were incubated at 37°C for 30 minutes, after which an SDS-PAGE loading buffer without DTT was added to stop the reaction and was loaded directly (without boiling) into an SDS-PAGE gel. Electrophoresis was performed at 4°C. The phosphorylated proteins were visualized with Phos-tag 300/460 Phosphoprotein Gel Stain (Perkin Elmer).

### Antibiotic determinations

To study the signal that triggers ACT production in the parent and mutant strains, the cells were grown at 30°C for four days as a lawn, inoculating 7,5×10^6^ spores/plate. The experiments were repeated twice and five different plates were used for each medium condition.

In the rest of the experiments, all the endogenous antibiotic production was observed on plates of solid medium inoculated with 10^5^ spores, either streaked out or added in a five-µL drop. The experiments were replicated at least twice on four different plates in each case. RED production was detected as the red colour of the colonies on PGA medium after 2 days. For ACT production, the strains were grown on NMMP at 30°C for at least 3 days to observe a blue halo around the colonies. For CDA production, the strains were grown on NA medium at 30°C for 2 days. Following this, the plates were overlaid with 5 mL of soft agar plus 60 mM Ca(NO_3_)_2_ inoculated with *Bacillus subtilis* as the test microorganism (0.2 mL, 0.25 OD) and incubated at 30°C for 20 hours. A replica plate without calcium was used as a negative control.

### Western blot assays

Total protein extracts were obtained from 5-mL samples (in triplicate) of the cultures. The proteins were resolved in SDS-PAGE (15% polyacrylamide in a MiniProtean II system, BioRad). After transfer to Immobilon-P (Millipore), the proteins were reacted with a pre-purified polyclonal 1∶50000 dilution of anti-XysA. XysA was detected by chemiluminescence with ECL western blotting detection reagents following the manufacturer's instructions (GE Healthcare), using horseradish peroxidase-coupled anti-rabbit secondary antibody.

### Oviedomycin production

The parental strain (M145) and Δ*abrA1/A2* were transformed with the cosmid CosAB4 containing the oviedomycin cluster and with empty cosmid pKC505 as a control (Table S2). Oviedomycin has a yellow colour that turns to orange-brown with time and is secreted to the culture medium. Oviedomycin production was analyzed on solid and in liquid NMMP media. On solid medium, 5-µL droplets containing 2.5×10^3^ spores of the corresponding strains were inoculated and the production of colored compounds was observed after growth at 30°C. In liquid medium, 10 mL of medium were inoculated with 4×10^5^ sp/mL of each strain and incubated at 30°C at 200 rpm for 5 days.

To perform bioassays against *Micrococcus luteus*, previous acetone extraction of solid cultures of the producer strains was carried out, the evaporated extract was resuspended in methanol, and 15 µL of this suspension was dropped onto a Whatman disc placed on a *M. luteus* lawn inoculated on YEPD. UPLC analysis for oviedomycin was performed as described in Mendez *et al.* [13].

## Results

### Which signal activates the AbrA system?

The AbrA1/A2 TCS is a pleiotropic negative regulatory system. The Δ*abrA1/A2* strain shows a significantly increased CDA, RED, and ACT production, and an accelerated morphogenetic program can also be observed in this strain. The increase in ACT production is mainly observed in NMMP medium and is nutrient-dependent [10]. Accordingly, some nutritional compound might be triggering HK auto-phosphorylation and the subsequent phosphate transfer to its cognate RR, repressing the antibiotic and differentiation pathways. When the nutrient becomes limiting, the pathway may be blocked and the de-phosphorylated RR may be unable to exert its repressing activity.

Since NMMP medium has a defined composition, the parent and mutant strains were grown as a lawn (see Materials and Methods) in complete and modified NMMP media lacking one of its components independently (phosphate buffer, glucose, (NH_4_)_2_SO_4_, MgSO_4_, casamino acids, or one or all of the four trace elements, respectively). The growth of both strains was monitored under all the different conditions, no differences being observed. The hypothesis was that if the nutrient signal were absent from the medium, the repressing effect exerted by AbrA2 would be prevented in the parental strain.

**Figure 1:**
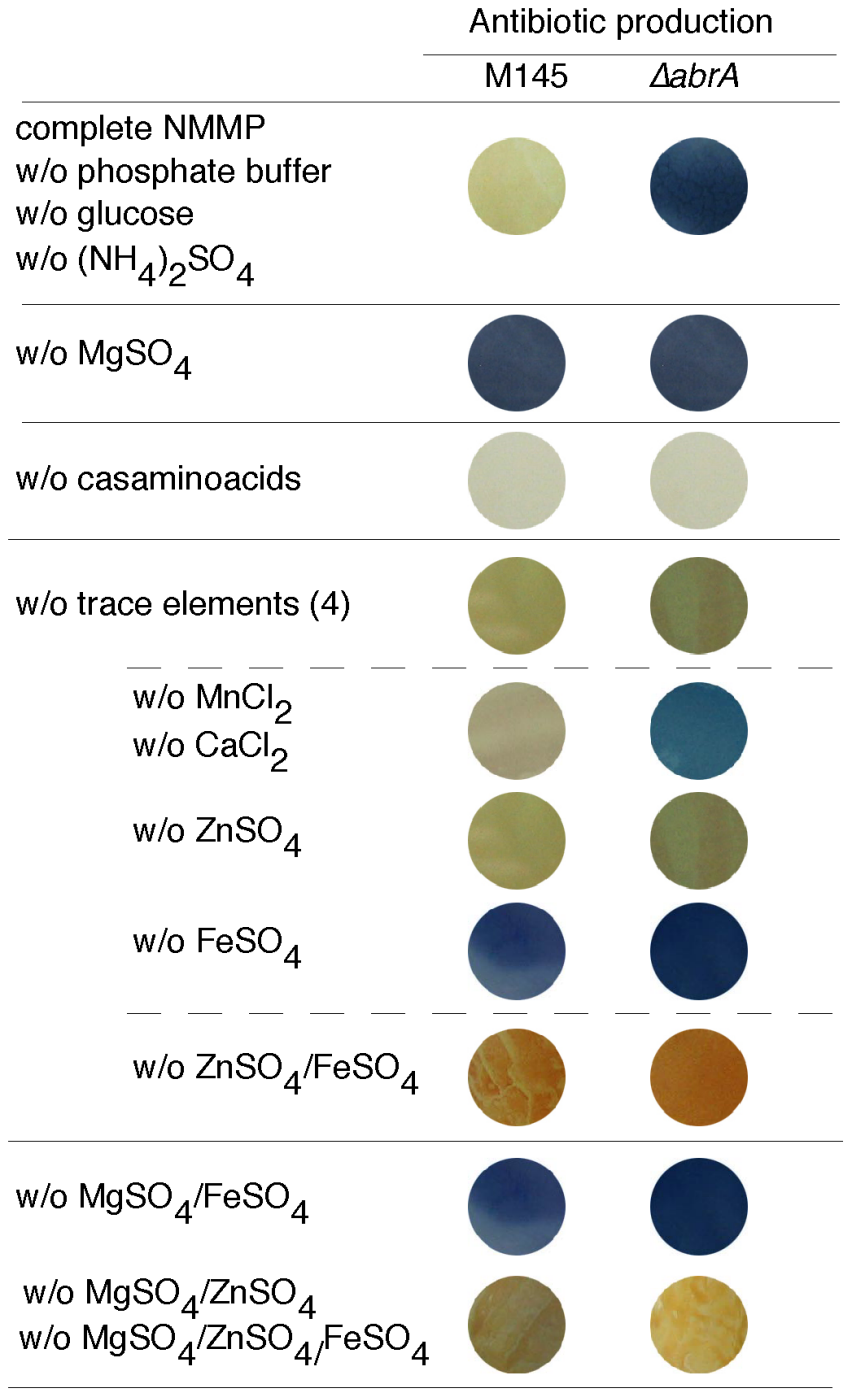
AbrA1 HK signal response in NMMP. ACT production (blue colour) of the parent strain (M145) and the mutant Δ*abrA1/A2* (Δ*abrA*) in NMMP complete medium and different modified versions lacking (w/o) one or several compounds of the original recipe. All plates were inoculated as a lawn with 7.5×10^6^ spores of the corresponding strains and the images correspond to backward sections of the plates after four days at 30°C.

Both strains were able to produce ACT at the same time and level when the medium lacked MgSO_4_. By contrast, the absence of casamino acids or the trace element solution prevented ACT production in both strains (Figure 1). A more detailed study involving the removal of each of the four trace elements separately demonstrated that this phenotype (non-ACT) was due to the absence of ZnSO_4_. Additionally, the elimination of only FeSO_4_ from the medium induced ACT production (de-repression) in the parent strain, similar to the absence of MgSO_4_ (Figure 1).

Thus, both casamino acids and ZnSO_4_ seemed to be essential for ACT production even in the Δ*abrA1/A2* strain. Zinc is a co-factor of some ACT biosynthetic enzymes such as cyclase/dehydratase SCO5090 [26] and some components of casamino acids are antibiotic precursors (ie. leucine, isoleucine, and valine) [27], and hence their absence could account for the absence of ACT production in both strains. However, both MgSO_4_ and FeSO_4_ needed to be present for the repression of ACT production to be maintained in the wild type, their effects not being additive (Figure 1: w/o MgSO_4_/FeSO_4_). Moreover, the phenotype of ZnSO_4_ absence prevailed over the one shown by the absence of FeSO_4_ or/and MgSO_4_, as expected (Figure 1).

Overall, these results offered us relevant information that narrowed down the activating signal(s) of AbrA system to two elements Mg and/or Fe. Whether this was a direct activation (signal sensed by the kinase) or indirect activation remains unknown.

### AbrA operon expression is auto-regulated and responds to Fe

The promoter functionality of the operon containing the AbrA TCS, *abrAp*, upstream of the *SCOs1742/43* genes, has been demonstrated previously in the complementation studies of Δ*abrA1/A2* [10]. As mentioned above, the expression of many TCS operons is auto-regulated by the induction/repression of the promoter through the binding of the phosphorylated RRs to their own promoters [9], [28].

**Figure 2:**
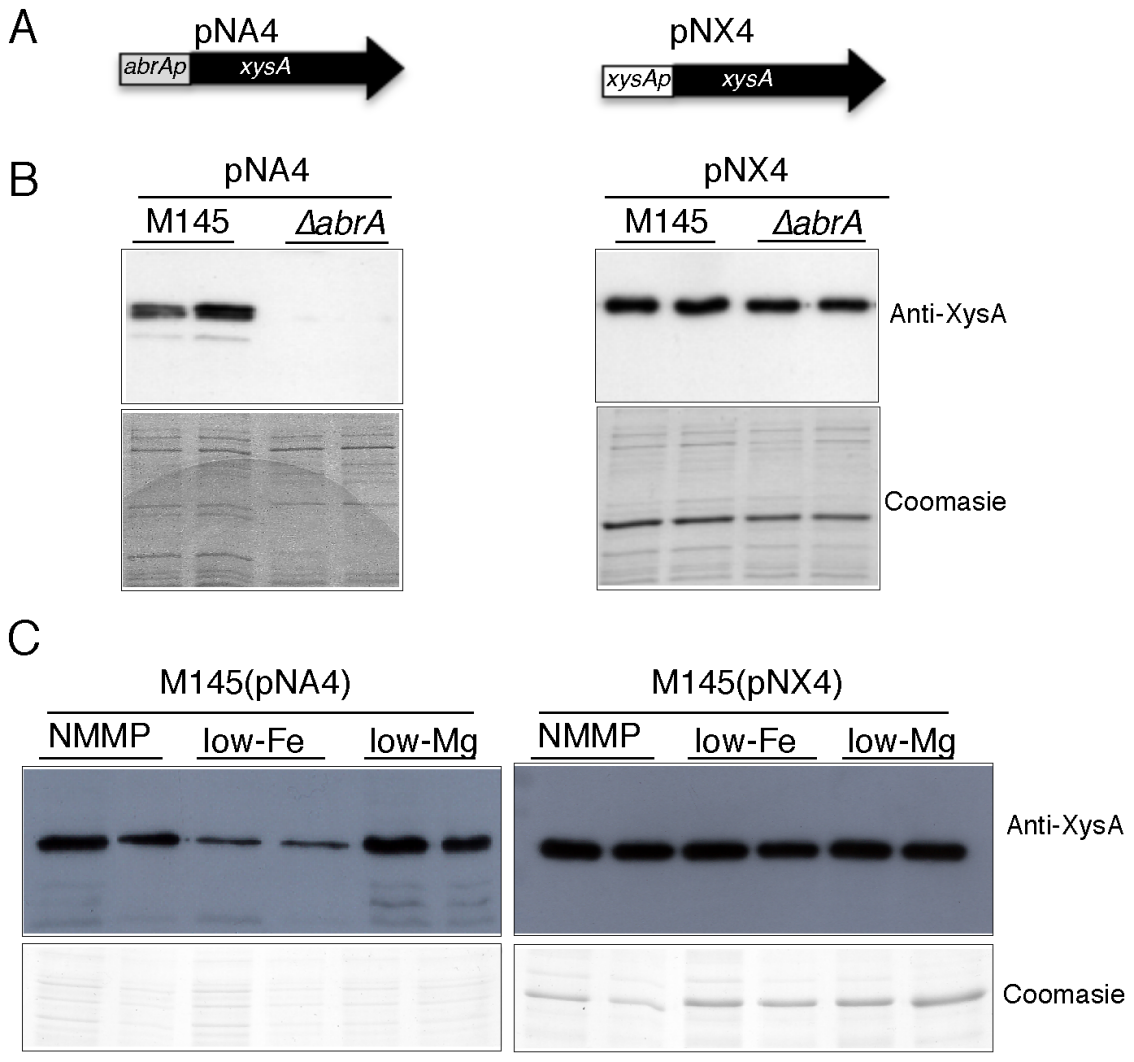
Auto-regulation of AbrA2 and dependence on Fe. A) Scheme of plasmids for xylanase gene expression (*xysA*) used as reporter. Plasmid pNA4: *xysA* under the control of *abrA* promoter (*abrAp*) was used, and plasmid pNX4: *xysA* under the control of its own promoter *xysAp* was used as a control. B) Top left panel: *xysA* expression triggered by pNA4 (*abrAp*) in the parent strain (M145) and the Δ*abrA1/A2* mutant (Δ*abrA*), respectively, is shown. Top right panel, control expression *by xysAp* (pNX4) in both strains is shown. C) Western blot showing *xysA* expression under *abrAp* (plasmid pNA4), or *xysAp*, (plasmid pNX4) in the parent strain M145 in different medium conditions: complete NMMP, low-Fe or low-Mg media. The xylanase signal corresponds to western blot assays using anti-xysA against supernatants of 7-day cultures in NMMP medium (100 µL of pNA4 and 2 µL of pNX4). In the bottom panels of B and C the amount of protein loaded in each lane is shown with a Coomassie blue stain. Two independent cultures of each construction are shown.

To test the hypothesis that *abrAp* may be auto-regulated by AbrA2-P, the multicopy plasmid (pNA4) was obtained. This plasmid contains as a reporter the *xysA* xylanase gene from *S. halstedii* JM8 under the control of *abrAp*. A plasmid carrying this *xysA* gene under its own promoter was used as a control of the experiment (pNX4) (Figure 2A). Xylanase production was studied in the parent strain and the null *abrA1/A2* strain, observing that under *abrAp* (pNA4) it only occurred in the wild-type strain at seven days culture (Figure 2B, left panel) although small production could be observed at the null *abrA1/A2* strain at longer incubation times. However, no significant differences in production were detected between strains when the control plasmid pNX4 was used at any time (Figure 2B, right panel). Accordingly, the activation of the operon expression is dependent on the presence of the *abrA1* and *abrA2* genes (Figure 2B left). Thus, the AbrA system positively regulates its operon promoter under these culture conditions directly through the phosphorylation of AbrA2 or indirectly through other as yet undetermined RRs.

The plasmids pNA4 and the control pNX4 were also used to study the putative signaling effect of Mg or Fe. The parent strain M145, harboring pNA4 or pNX4, respectively, was grown in NMMP complete medium and in derivative media with only 10 µM FeSO_4_ (low-Fe) or 10 µM MgSO_4_ (low-Mg). In liquid cultures, total absence of these compounds did not permit growth at all of the strains even without the plasmids and consequently there was no antibiotic production (Figure S1). As mentioned above, there was normal growth observed on solid medium in these conditions (w/o Fe or w/o Mg), meaning that the trace elements present in the agar must be sufficient to allow normal growth.

As shown in Figure 2C the production of xylanase with *abrAp* (pNA4) in low-Fe conditions was considerably reduced with respect to that obtained with complete NMMP medium or in medium with low Mg. The observed growth of both strains was similar under all the different conditions assayed and when the *xysp* promoter supported production (pNX4), a similar production was obtained in all media (Figure 2C, right panel). Thus, iron seems to act as a signal to activate the expression of the reporter under *abrAp*. A low-level production of xylanase was observed with plasmid pNA4, probably because the compound was not completely absent. Our hypothesis is that under this condition (low-Fe) the histidine kinase AbrA1 is mainly unphosphorylated and therefore the RR AbrA2 is in its inactive unphosphorylated form and is unable to bind to *abrAp* and trigger the same level of expression of the reporter gene as in the NMMP complete medium. By contrast, the absence of Mg did not correlate with a diminished production of the reporter. Since Mg is necessary for the proper functioning of the TCS, we suggest that the small amount of this element added to the medium (10 µM) might be sufficient for TCS functionality. In light of these results, we propose that Fe could be the activating signal of the TCS AbrA1/A2 in NMMP medium.

### The toxicity of AbrA2 overexpression is dependent on its phosphorylation state

**Figure 3:**
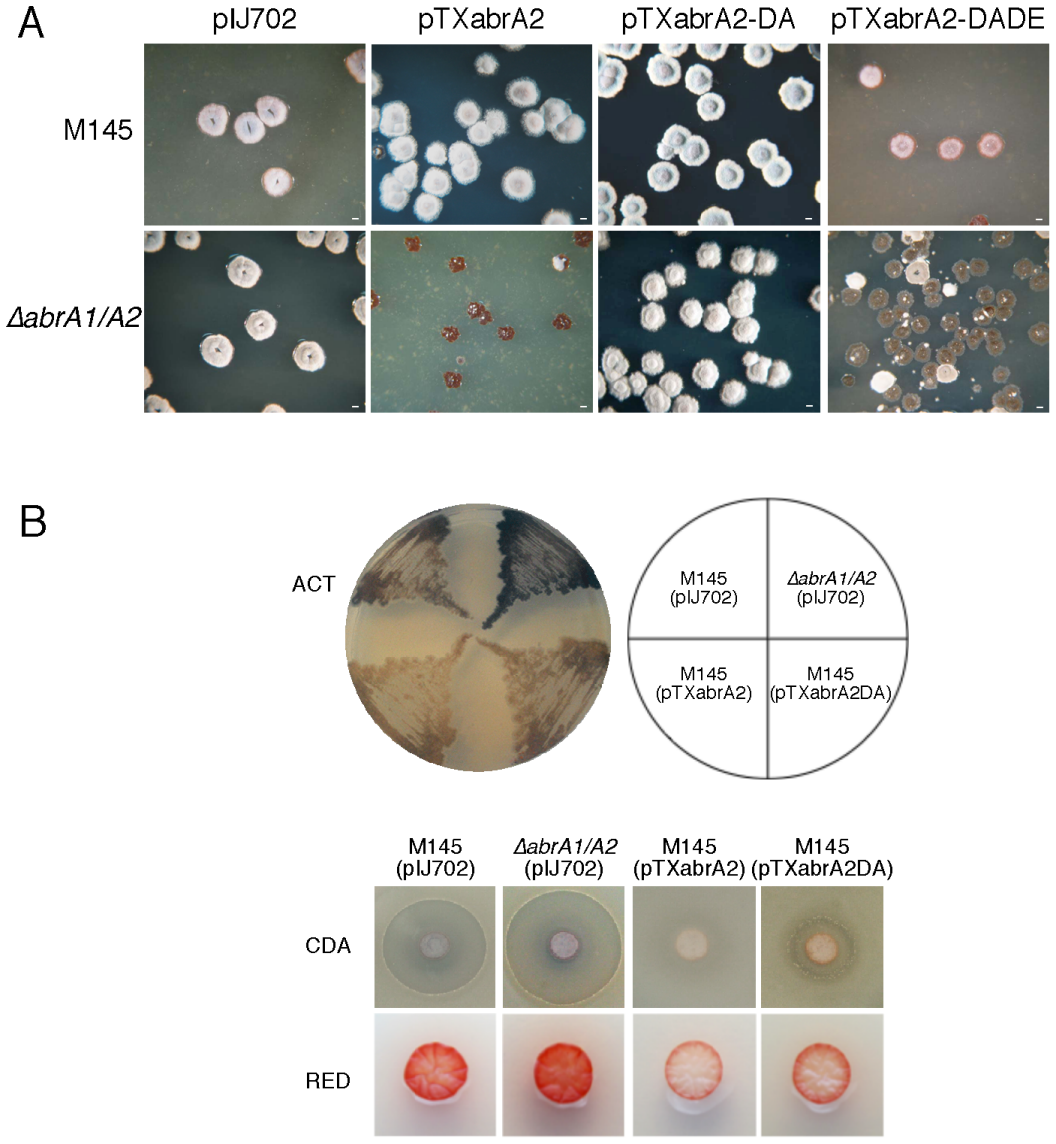
Overexpression of *abrA2*. A) Colony morphology of *S. coelicolor* M145 and *S. coelicolor ΔabrA1/A2* strains transformed with different multicopy plasmids: pIJ702 (control), pTXabrA2 (expressing the AbrA2 RR under the control of *xysAp*), and its derivatives pTXabrA2-DA (D_55_A) and pTXabrA2-DADE (D_10_A, D_55_E). The photographs correspond to four-day cultures on R2(YE) medium. Bar: 1 mm. B) Phenotypes of AbrA2 overexpression in *S. coelicolor* M145 compared to the mutant Δ*abrA1/A2* phenotype. The phosphoablative version (pTXabrA2-DA) was included in the study. Upper part: production of ACT in NMMP medium plates (72 h); middle part: CDA bioassay against *B. subtilis* at 48 h; lower part, RED production at 48 h.

The effect of the expression of multiple copies of *abrA2* in *S. coelicolor* M145 and in the Δ*abrA1/A2* strains was studied using the plasmid pTXabrA2, in which the promoter *xysAp* controls *abrA2* expression. In both strains the transformation efficiency of pTXabrA2 was similar to that of the empty vector used as a control, pIJ702. Nevertheless, the colonies obtained in the Δ*abrA1/A2* strain exhibited a slow and aberrant morphological development while in the parent strain the transformed colonies had a healthy phenotype (Figure 3A). Moreover, the aberrant Δ*abrA1/A2* (pTXabrA2) colonies grew poorly when they were re-inoculated onto plates of fresh medium or in liquid medium.

To study whether the selective toxicity in the Δ*abrA1/A2* (pTXabrA2) strain was due to the phosphorylated form of AbrA2 despite the absence of its AbrA1 HK, an AbrA2 phosphoablative mutant derivative protein was constructed. The putative Asp residue involved in phosphorylation, Asp55, was replaced by an Ala. Overexpression of this variant, AbrA2-DA, did not elicit toxicity either in the wild-type or in the double mutant, showing the importance of the phosphorylation state of AbrA2 as regards toxicity (Figure 3A). Once it had been confirmed that this toxic effect was caused by a hyperactivation of the RR (an excess of phosphorylated RR), we decided to use this phenotype as readout to follow the activation of AbrA2 in different contexts. Taken together, the results were consistent with the notion that AbrA2 phosphorylation still occurred when its cognate HK AbrA1 was not present. Remarkably, the fact that such overexpression was not toxic in the parent strain M145(pTXAbrA2), in which the kinase AbrA1 was present, suggested that this HK might also be controlling the phosphorylation state of AbrA2 by intrinsic phosphatase activity, as described in other HKs [9].

To test this hypothesis, an *abrA2* phosphomimetic mutant was generated with the expectation that its expression would be toxic even when overexpressed in the parent strain. To do so, another Asp (Asp-10) of the acidic pocket was replaced by Ala, and the Asp-55 was replaced by Glu (AbrA2-DADE). As shown in Figure 3A, the overexpression of AbrA2-DADE was toxic in both strains (parent strain and double mutant). A mild toxicity phenotype was observed in the wild-type strain, possibly due to the presence of an unmodified copy of *abrA2* in the genome. This result supported the idea that the AbrA1 HK might be responsible for the phosphorylation (by phosphotransfer) and dephosphorylation (by phosphatase activity) of AbrA2, maintaining a balance between non-phosphorylated and phosphorylated RR at each moment.

Regarding antibiotic production, the overexpression of AbrA2 in the parent strain, which was not toxic, led to the opposite phenotypes to those observed in the absence of these genes in Δ*abrA1/A2*. Strain M145 (pTXAbrA2) produced lesser antibiotic (ACT, RED and CDA) than the strain carrying the empty vector pIJ702, in contrast to the enhanced antibiotic production of the Δ*abrA1/A2* (pIJ702) mutant (Figure 3B). The wild-type strain transformed with the phosphoablative mutant showed an intermediate phenotype between those obtained with the empty and pTXAbrA2 plasmids due to a remaining unmodified copy of the *abrA2* in the genome.

The individual *S. coelicolor ΔabrA1* and *S. coelicolor ΔabrA2* strains were generated using the PCR-targeting approach described by Gust *et al.* [19] (see Materials and Methods). Phenotypic analysis of the growth and antibiotic production of the parent strain (M145 strain), Δ*abrA1, ΔabrA2* and the double mutant Δ*abrA1/A2*, previously described [10], revealed that the phenotypes (antibiotic production and morphological differentiation) of individual deletion of the AbrA TCS genes were intermediate between the parent and double mutant strain in NMMP medium (Figure S2A). These phenotypes were complemented by the pHabrA1 and pHabrA2 plasmids. Transformation of these strains, Δ*abrA1* and Δ*abrA2*, with pTXabrA2 and with the control pIJ702 demonstrated the toxicity of the overexpression of AbrA2 in the Δ*abrA1* strain but not in the Δ*abrA2* strain, which has a functional AbrA1 kinase (Figure S2B). This again points to other kinases or regulators cross-talking.

### Small phospho-donors are not able to phosphorylate AbrA2 *in vitro*

As seen in the overexpression experiments, the phosphorylation of AbrA2 was necessary for it to exert its toxicity and took place even in the absence of the cognate kinase. A possible explanation for this could be phosphate transfer from small intracellular phospho-donors, such as acetyl phosphate, to the RR, as reported in other systems [8].

**Figure 4:**
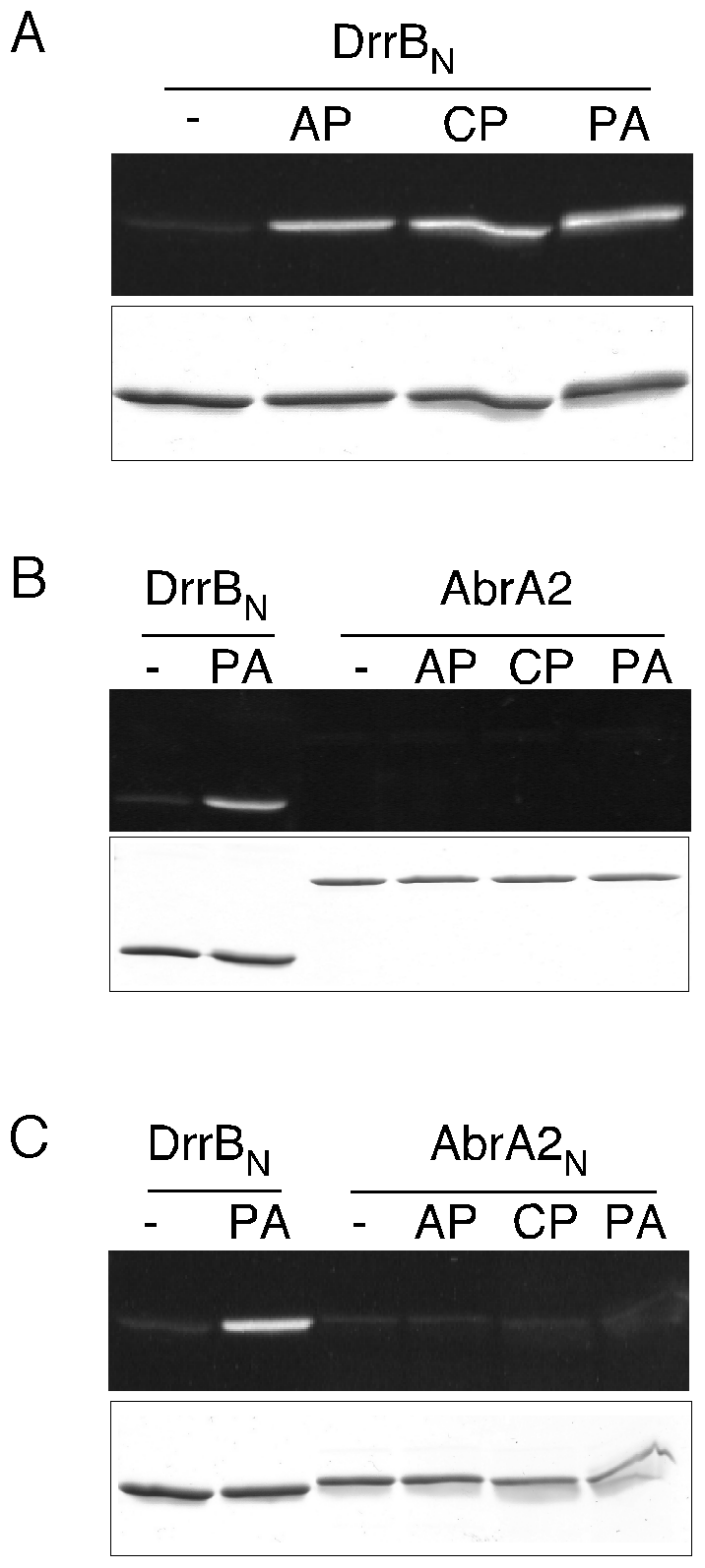
Phosphorylation assays with small phospho-donors. Phosphorylation of DrrB_N_ (A), AbrA2 (B) or AbrA2_N_ (C) proteins with acetylphosphate (AP), carbamoylphosphate (CP) or phosphoramidate (PA) visualized with the Phos-tag stain in SDS-PAGE (“-” means no phospho-donor added). In the bottom part of each panel the amount of protein used is shown with a Coomassie blue stain.

The AbrA2 protein with a His(6) tag was expressed and purified in *E. coli* and used as a substrate in *in vitro* phosphorylation assays by several potential phospho-donors, using a non-denaturing PAGE procedure. Acetyl phosphate, carbamoylphosphate and phosphoramidate were used as phospho-donors (see Materials and Methods) and the protein DrrB_N_ from *Thermotoga maritima* was used as a positive control [24]. The phosphorylated proteins were visualized using a specific phosphorylation stain (Phos-tag). The three compounds tested were able to phosphorylate the control protein DrrB_N_ while no phosphorylation band was observed when AbrA2 was used (Figure 4A and B). It is possible that the receptor domain might not be accessible, having a closed conformation with the effector domain through a domain-domain interaction, as occurs in other regulators [24], [29]. To study this possibility, a truncated AbrA2 form was generated. The amino terminal domain AbrA2_N_ (corresponding to the first 141 amino acids) was expressed and purified, but the results using this receiver domain as a substrate were also negative (Figure 4C).

Therefore, none of the phospho-donors assayed was responsible, at least *in vitro*, for AbrA2 phosphorylation. These results suggested that AbrA2 phosphorylation in the absence of the AbrA1 kinase was likely a consequence of other kinases that phosphorylate AbrA2 non-specifically by cross-talking.

### Role of the ABC transport system of the SCO*1742/45* operon in antibiotic production

The ABC transporter system encoded directly upstream of the two-component system on the *S. coelicolor* chromosome exhibits sequence similarity to antibiotic and multidrug resistance (MDR) export transporters. It is encoded by two genes, (SCO*1742/43*), one coding for a nucleotide-binding domain (NBD) and the other a transmembrane domain (TMD). The NBD of the first gene displays similarities to MDR transporters and to the NBD BcrA of the *Bacillus licheniformis* ABC transporter BcrABC, which is involved in the transport of bacitracin [30]. In order to investigate its role in antibiotic production and possible interactions with the two-component system of the operon, two mutants were constructed using the REDIRECT technology (see Materials and Methods). In one of them, only the two ABC transporter genes were deleted (*S. coelicolor Δ1742/43*). In the other, all four genes of the operon were deleted (*S. coelicolor Δ1742-45)*.

**Figure 5:**
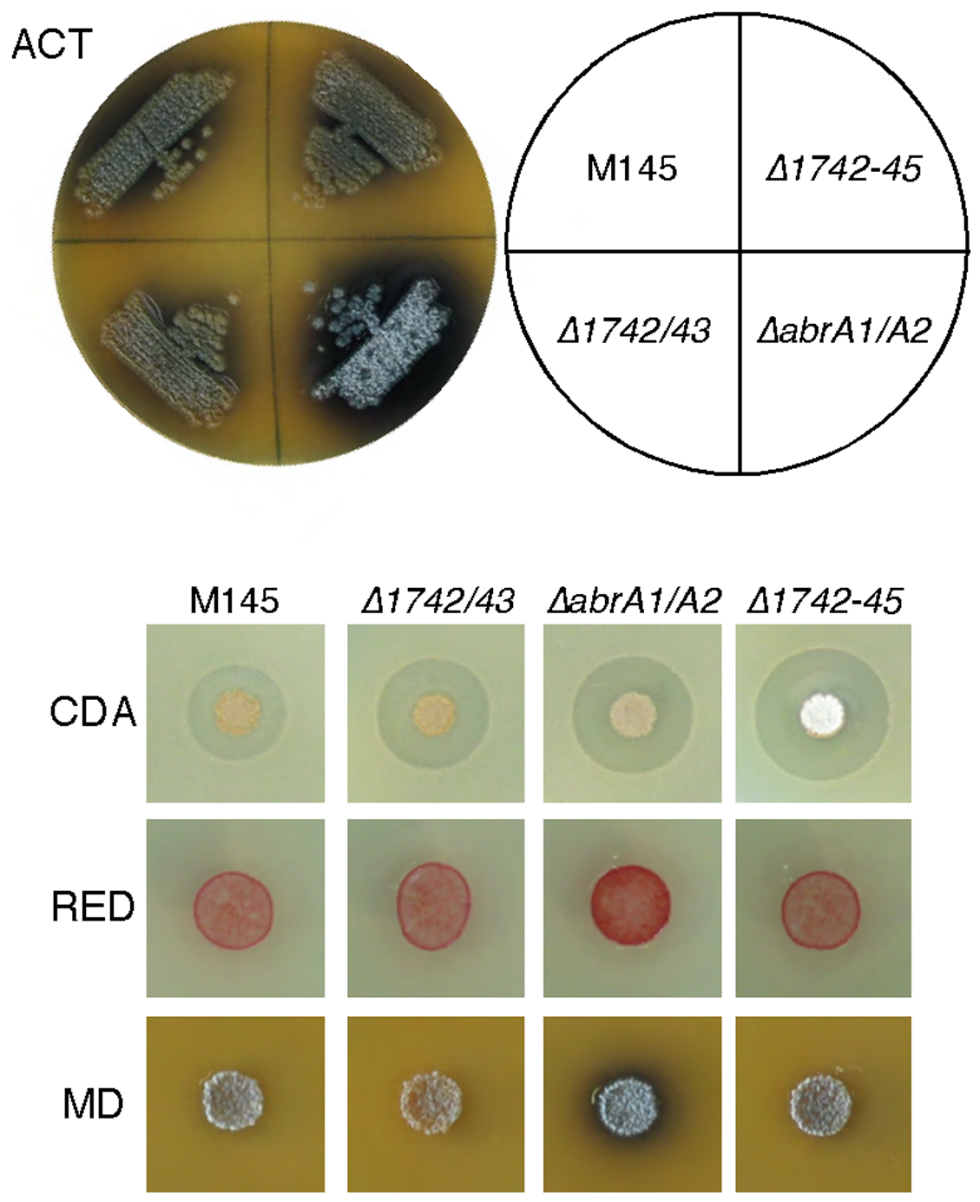
Antibiotic production and morphological differentiation of different *SCO1742-45* operon mutants. Production of ACT on NMMP at six days of growth; CDA at two days of growth in NA medium; RED at two days in PGA, and morphological differentiation at six days of growth in MSA.

Deletion of the *SCO1742* and *SCO1743* genes (Δ*1742/43*) did not elicit the early sporulation observed in the Δ*abrA1/A2* strain, and deletion of the whole operon (Δ*1742-45*) resulted in a suppression of the Δ*abrA1/A2* phenotype (Figure 5). This suppression was also observed in ACT and RED production. Nevertheless, similar to Δ*abrA1/A2*, both new mutants produced higher amounts of CDA on solid NA medium as compared to the parent strain, the highest CDA production being observed in the Δ*1742-45* mutant.

### The Δ*abrA1/A2* strain as a heterologous host for antibiotic production

Since AbrA1/A2 was involved in the negative regulation of antibiotic production we wondered whether the null strain Δ*abrA1/A2* might be a good candidate for the heterologous expression of antibiotic clusters from other organisms. The oviedomycin biosynthetic gene cluster from *S. antibioticus* ATCC 11891 [11], [12], [13] was used to check this possibility. The cosmids containing the oviedomycin cluster (CosAB4) and the empty one (pKC505) were introduced in the parental and mutant strains, M145 and Δ*abrA1/A2*, and production of this compound was checked in solid and liquid NMMP media (see Materials and Methods).

**Figure 6:**
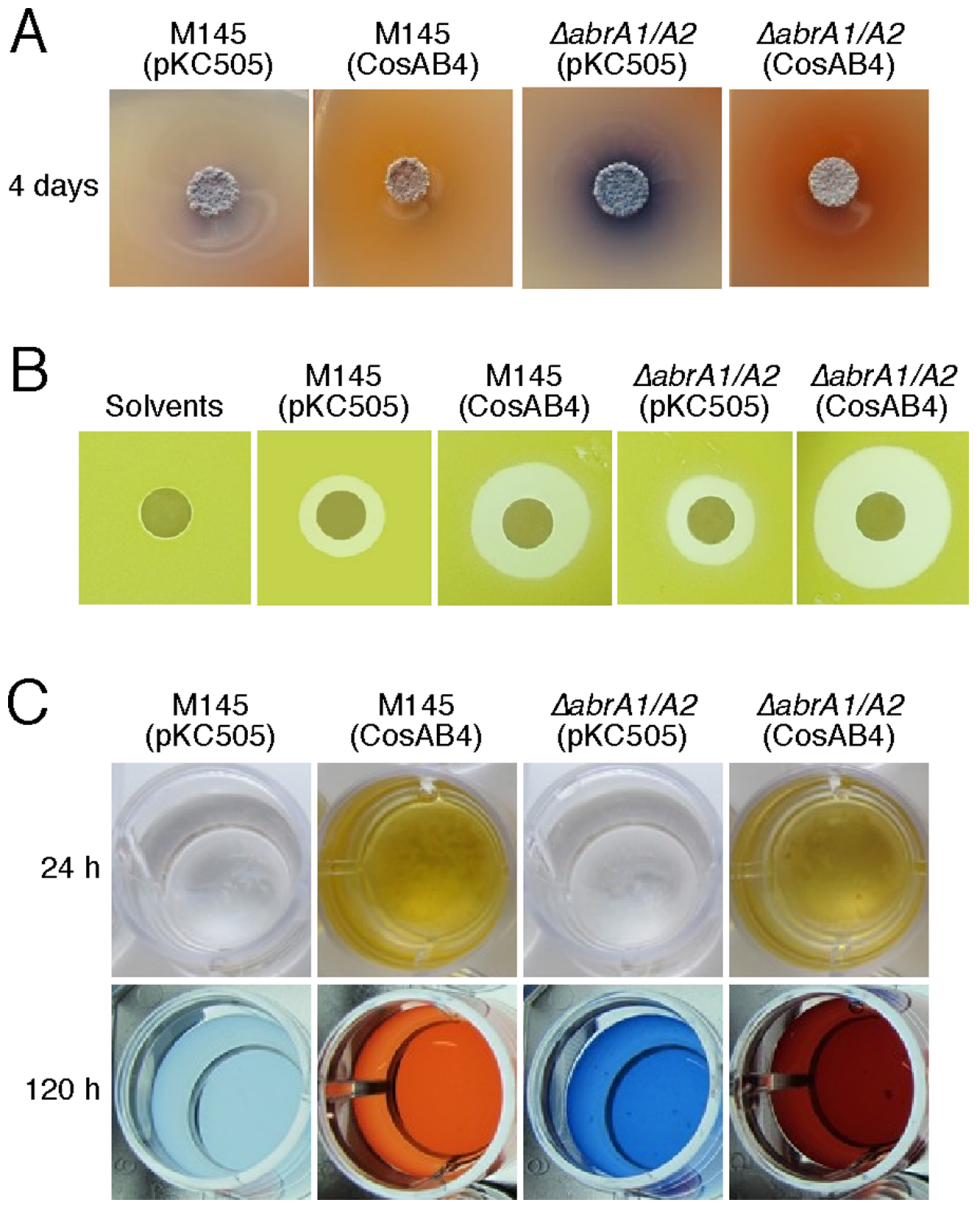
Oviedomycin production. Oviedomycin production of M145 and Δ*abrA1/A2* strains carrying the cosmid CosAB4, containing the oviedomycin cluster, or an empty control pKC505. A) Solid NMMP medium at 4 days of growth. B) Antibiotic activity against *M. luteus* of acetone-methanol (solvents) extracts from the different strains grown for 6 days on solid NMMP. C) Supernatant of liquid NMMP cultures at 24 and 120 hours of growth.

As observed in Figure 6A, a brown-reddish compound corresponding to oviedomycin was produced in both strains on solid medium, and the production by the Δ*abrA1/A2* mutant was higher than in M145. Moreover, bioassays against *M. luteus* showed that the product was functional and that the strain with the greatest growth-inhibiting potential was Δ*abrA1/A2* (CosAB4) (Figure 6B). Production in liquid medium was also assessed and the same result was observed (Figure 6C). Interestingly, the production of oviedomycin occurred earlier in time and even displaced the ACT production observed in the strains carrying the empty cosmid in both solid and liquid media (blue colour). UPLC assays showed that the colour observed in the strains carrying CosAB4 corresponded to oviedomycin and that the mutant strain produced double amount of oviedomycin as compared with M145 (Figure S3).

Accordingly, the antitumoral angucyclinone polyketide oviedomycin was produced heterologously in *S. coelicolor* M145, with a significantly increased efficiency in the mutant strain lacking the TCS AbrA1/A2.

## Discussion

The negative antibiotic regulator TCS AbrA1/A2 (AbrA), also involved in morphological differentiation [10], forms part of an operon with two genes that constitute an ABC transporter system, one encoding an ATPase and the other a permease with similarity to the BcrAB-type transporters described for firmicutes [31]. The operon has positive auto-regulation through the AbrA system. Its expression activation is dependent on the presence of the *abrA1* and *abrA2* genes (Figure 2B left). Although the involvement of transcriptional regulators other than AbrA2 cannot be ruled out, based on the conventional mode of action of TCSs it seems likely that the RR would control its own expression. However, a basal expression of the genes explains the phenotypes observed in this work with the different mutants obtained. In the Δ*1742/43* strain, lacking the ABC system, the level of antibiotics and morphological differentiation were similar to those of the parent strain, because the TCS that controls these pathways was functioning at its normal level as shown by q-RT-PCR (data not shown). Nevertheless, when only the *abrA1/A2* genes were absent, an increased level of ACT and RED production and an accelerated differentiation were observed, whereas in the strain lacking the four genes (Δ*1742-45*) these phenotypes were suppressed. As expected, the basal expression of ABC genes in the Δ*abrA1/A2* strain was corroborated by RT-PCR (Figure S4). This suggests that there is an essential role of ABC genes in the transport of compounds necessary for the observed antibiotic overexpression, and that a basal expression is sufficient for this function to be performed. In fact, on the *S. coelicolor* chromosome genes with related function are frequently arranged adjacent to each other [32]. Thus, a possible explanation might be that the ABC transporter could be involved in the expulsion of harmful or toxic by-products coming from the biosynthesis of these antibiotics or one of the precursors needed. Efflux pumps for an antibiotic itself however, are more likely to lie directly within the respective biosynthetic gene cluster, as demonstrated for the ACT cluster [33], [34].

A study carried out on the major facilitator superfamily putative MDR transporter EbrC in *S. lividans* demonstrated that its deletion leads to retardation in morphological differentiation on specific media, whereas its natural role in drug resistance remains unclear [35]. These authors therefore proposed that the actual role of most MDR transporters in streptomycetes would consist of the export of toxic intermediate catabolic products from the cell, rather than of the efflux of externally added drugs. However, it is important to note that in our study the deletion of the ABC transporter in the mutant Δ*1742/43* did not negatively influence differentiation on the solid media used. Instead, its deletion in the mutant Δ*1742-45* somehow prevented an acceleration of differentiation, as noted for the mutant Δ*abrA1/A2*.

On the other hand, we observed that when AbrA2 was overexpressed in a mutant lacking the kinase gene, the phosphorylation of AbrA2 was responsible for a toxic phenotype since a phosphoablative mutant did not produce it. Crosstalk from other kinases might explain the activation of AbrA2 in the absence of AbrA1; additionally, phosphorylation by low-molecular weight phospho-donors *in vivo* could provide an additional mechanism for this activation. Nevertheless, this phosphorylation was not observed *in vitro* when small phospho-donors were used in the reaction. The phenotypes of the kinase mutants of other different TCSs have also shown that response regulators such as CheY and CheB can be phosphorylated in the absence of their cognate kinases [8]. The intermediate phenotype of the Δ*abrA1* with respect to the parent and double mutant strains also corroborates an unspecific, less efficient phosphorylation of AbrA2 by other kinases in the absence of AbrA1. Cross-talk in the other direction, meaning that the AbrA1 HK phosphorylates other RRs in the absence of AbrA2, can also be considered, because the single mutant lacking the AbrA2 RR, Δ*abrA2*, also had an intermediate phenotype.

Due that the toxic effect was not observed in the parent strain we propose that the kinase AbrA1 might be controlling the phosphorylation state of the RR by adjusting the long-term steady-state levels of activated molecules by de-phosphorylation and therefore that this control could prevent the toxicity of AbrA2 overexpression in the parent strain. Histidine-kinases frequently function as phosphatase-activating proteins to facilitate the de-phosphorylation of response regulators. In some cases, this may be their principal regulatory role [8]. The AbrA1 phosphatase control would not occur in the Δ*abrA1/A2* or Δ*abrA1* strains, in which the phosphorylation must be carried out by other kinases or phospho-donors and therefore the presence -due to overexpression- of more activated AbrA2-P molecules could cause toxicity. In conclusion, it seems that there are alternative phosphorylation mechanisms of AbrA2 but no alternative de-phosphorylation mechanisms.

A detailed study of the components of the defined medium NMMP in which TCS null-mutant phenotypes were observed allowed us to determine that the presence of Fe and Mg was necessary for the repression of antibiotic production mediated to be maintained. Besides, the auto-positive activation of the operon promoter of AbrA system responds directly or indirectly to the presence of Fe, as also corroborated using the *xysA*-reporter plasmid pNA4. In contrast, Mg does not seem to be important for activation of the expression of AbrA system in the promoter activity triggered by this system. Since polyketide synthesis does not require the presence of iron, iron deficiency may represent a significant regulation factor. In fact, Coisne *et al.* reported that ACT was produced early on a defined culture medium under conditions of iron deficiency and that the excretion of ACT appeared to be closely related to iron deficiency [36]. Because there is no iron-binding domain (hemo), detected with HemeBIND software [37], in the structure of the AbrA1 HK sensor domain, an auxiliary protein could play a role in this binding, such as in the HbpS-SenS-SenR system of *S. reticuli* [38]. Alternatively, a more complex network containing other TCSs could be involved, such as the PhoP/Q- PmrA/B interactions described in *Salmonella* [39], [40]. More studies are needed to determine how this metal mediates the AbrA signaling pathway.

Finally, the capacity of the strain lacking the TCS system, Δ*abrA1/A2*, to overproduce oviedomycin, an angucyclinone polyketide produced by *S.antibioticus* ATCC 11891, has been demonstrated. This antibiotic shows antitumor activity *in vitro* and induces apoptosis in cancer cell lines. Use of the genetically modified strain described here as a heterologous host could contribute to the production of new antibiotics or to improvements in the production efficiency of compounds from species that are not so effective at producing their own secondary metabolites, thus exploiting the “hidden biosynthetic” potential available in all *Streptomyces* genomes [41], [42].

## Supporting Information

Figure S1 **Liquid cultures in NMMP of** ***S. coelicolor*** **M145 and** ***S. coelicolor *Δ*abrA1/A2*** **with low or without Fe or Mg.**
(PDF)

Figure S2 **Phenotypes of** ****Δ*abrA1*** **and** ****Δ*abrA2*** **single mutants.**
(PDF)

Figure S3 **Oviedomycin UPLC profiles production.**
(PDF)

Figure S4 **Expression of** ***SCO1742*** **in the different strains by RT-PCR.**
(PDF)

Table S1 **Primers used in this work.**
(PDF)

Table S2 **Plasmids and cosmids used in this work.**
(PDF)
