# Supplementary material for: Regulation of the AbrA1/A2 Two-Component System in Streptomyces coelicolor and the Potential of Its Deletion Strain as a Heterologous Host for Antibiotic Production
Source: PLoS One. 2014 Oct 10;9(10):e109844. doi: 10.1371/journal.pone.0109844 (PMC4193843; doi:10.1371/journal.pone.0109844)
Supplement: Figure S1 — Liquid cultures in NMMP of S. coelicolor M145 and S. coelicolor ΔabrA1/A2 with low or without Fe or Mg. (PDF) [file pone.0109844.s001.pdf]

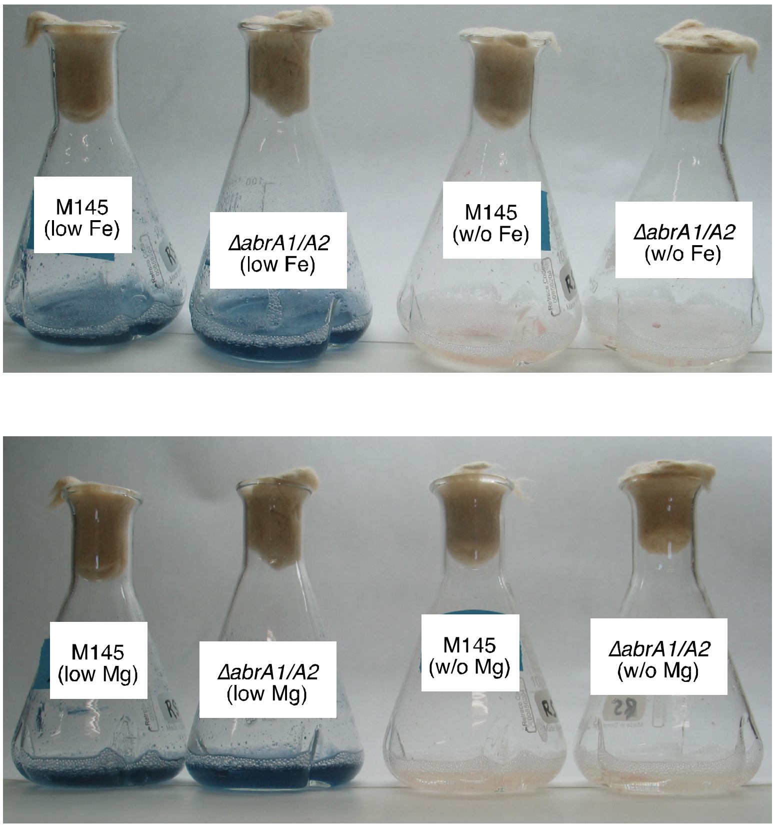

Figure S1: Liquid cultures of *S. coelicolor* M145 and *S. coelicolor* Δ*abrA1/A2* in NMMP with low or without Fe or Mg.
Upper part: NMMP medium with 10 μM Fe (low Fe) or without Fe (w/o Fe)
Lower part: NMMP medium with 10 μM Mg (low Mg) or without Mg (w/o Mg)
