# Supplementary material for: Regulation of the AbrA1/A2 Two-Component System in Streptomyces coelicolor and the Potential of Its Deletion Strain as a Heterologous Host for Antibiotic Production
Source: PLoS One. 2014 Oct 10;9(10):e109844. doi: 10.1371/journal.pone.0109844 (PMC4193843; doi:10.1371/journal.pone.0109844)
Supplement: Figure S2 — Phenotypes of ΔabrA1 and ΔabrA2 single mutants. (PDF) [file pone.0109844.s002.pdf]

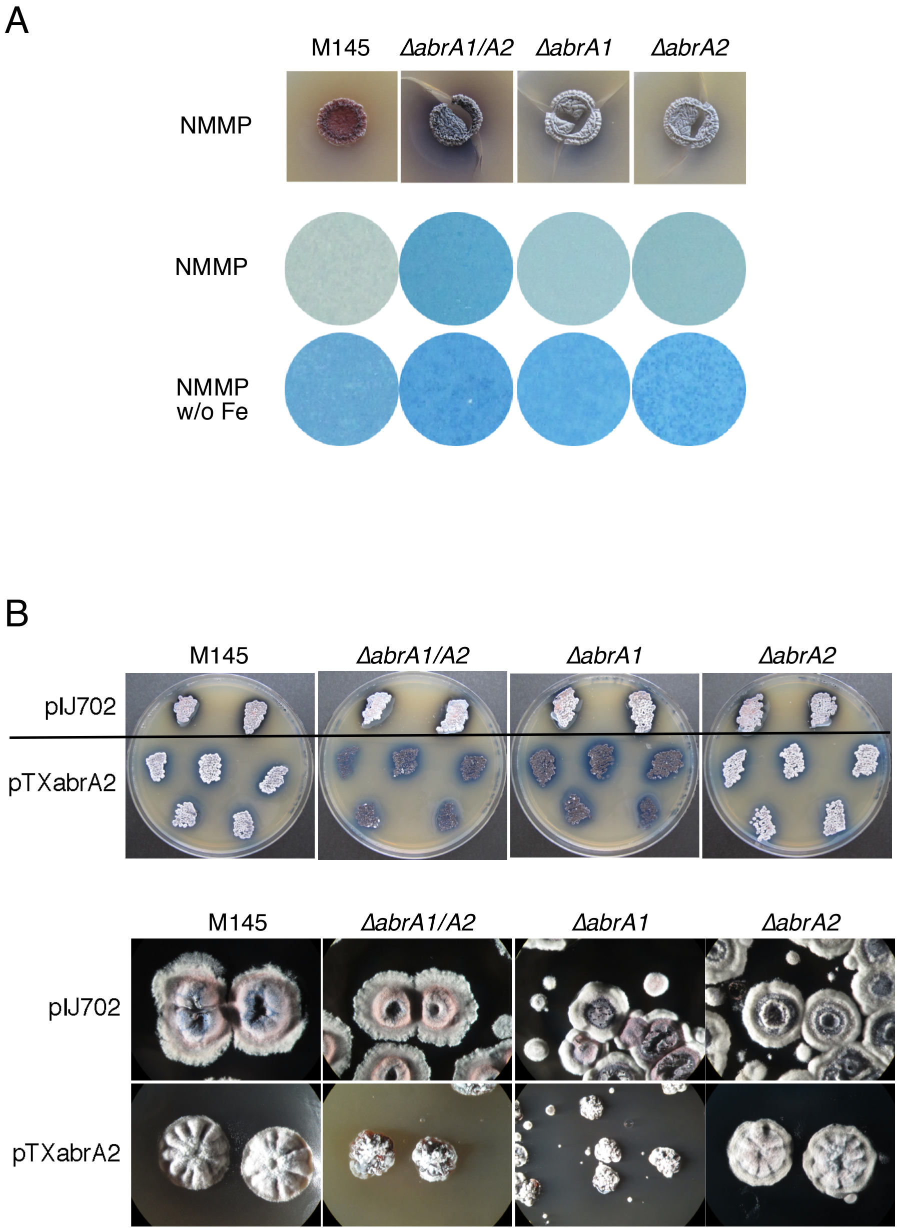

Figure S2: Phenotypes of Δ*abrA1* and Δ*abrA2* mutants.

A) ACT production and differentiation of colonies of the different strains (upper part) in NMMP at seven days (upper part); in the lower part, ACT production of the strains inoculated as a lawn in NMMP with or without (w/o Fe) at three days.and the images correspond to backward sections of the plates.

B) Morphology of *S. coelicolo*r M145 , Δ*abrA1/A2*, Δ*abrA1*, and Δ*abrA2* strains transformed with pIJ702 (control) or pTXabrA2 (expressing the AbrA2 RR under the control of *xysAp*). The photographs correspond to four-day cultures on R2(YE) medium. Upper part: growth in patches of different transformants; lower part: colony growth morphology.
