# Supplementary material for: Regulation of the AbrA1/A2 Two-Component System in Streptomyces coelicolor and the Potential of Its Deletion Strain as a Heterologous Host for Antibiotic Production
Source: PLoS One. 2014 Oct 10;9(10):e109844. doi: 10.1371/journal.pone.0109844 (PMC4193843; doi:10.1371/journal.pone.0109844)
Supplement: Figure S3 — Oviedomycin UPLC profiles production. (PDF) [file pone.0109844.s003.pdf]

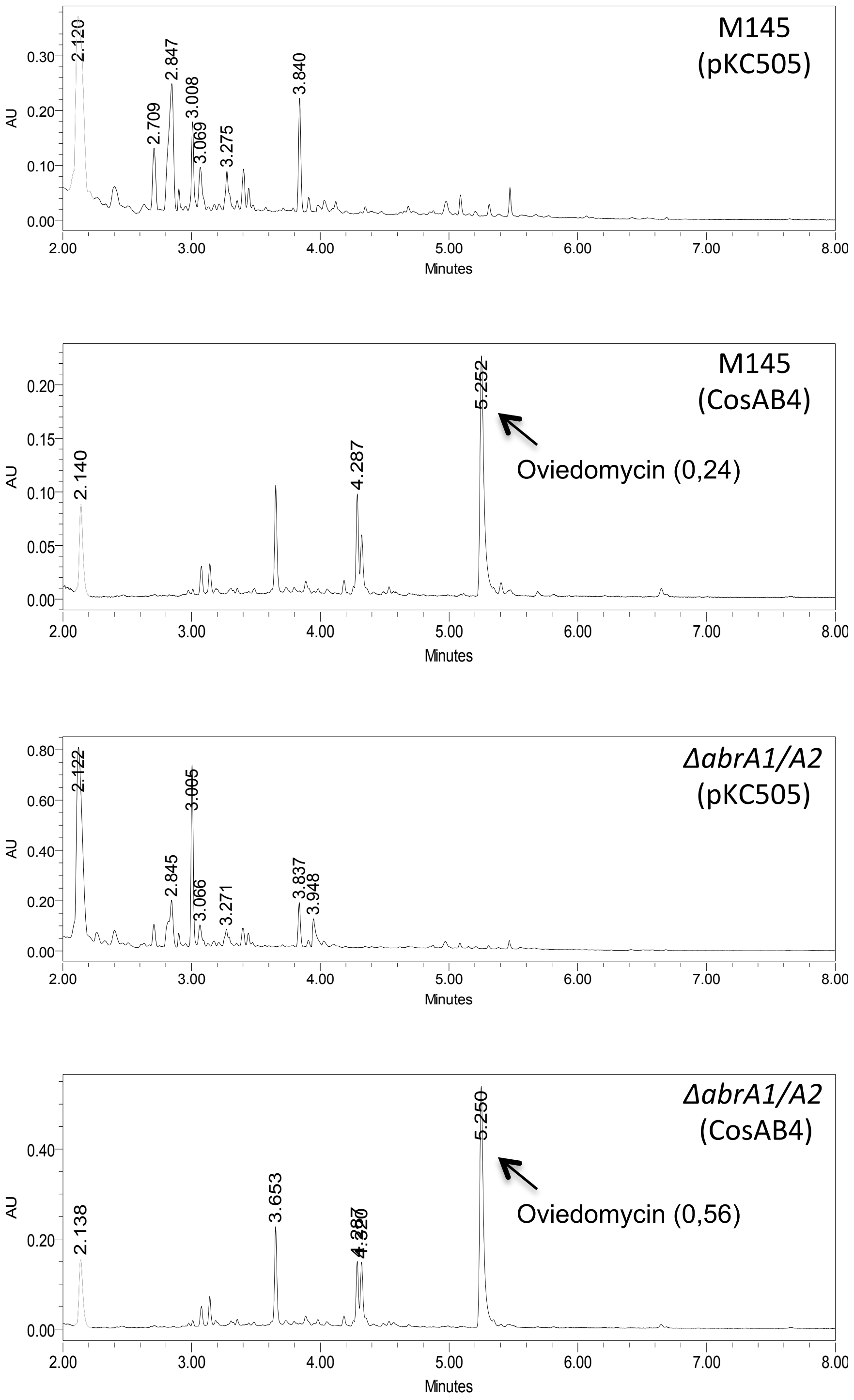

Figure S3: Oviedomycin UPLC profiles production.
UPLC profiles of *S. coelicolor* M145 and *S. coelicolor* Δ*abrA1/A2* harbouring pKC505 (empty vector) or CosAB4 (containing oviedomycin cluster) Oviedomycin peak is marked with an arrow and the peak height is shown in brackets
