# Supplementary material for: Regulation of the AbrA1/A2 Two-Component System in Streptomyces coelicolor and the Potential of Its Deletion Strain as a Heterologous Host for Antibiotic Production
Source: PLoS One. 2014 Oct 10;9(10):e109844. doi: 10.1371/journal.pone.0109844 (PMC4193843; doi:10.1371/journal.pone.0109844)
Supplement: Figure S4 — Expression of SCO1742 in the different strains by RT-PCR. (PDF) [file pone.0109844.s004.pdf]

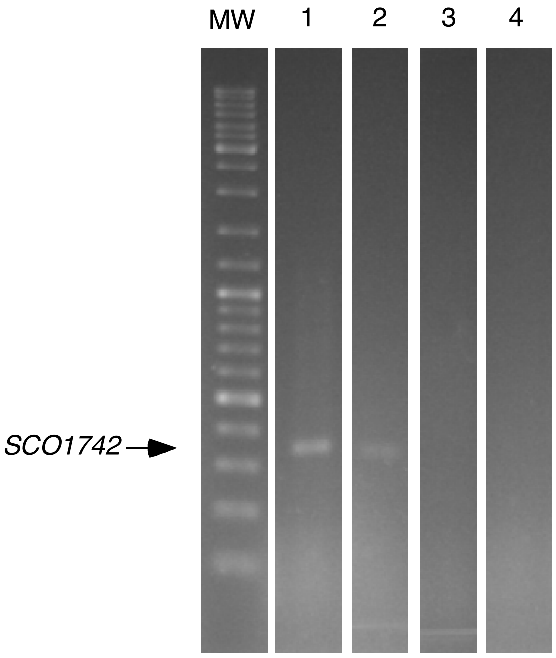

MW: Molecular weight ladder

1: *S. coelicolor* M145

2: *S. coelicolor ΔabrA1/A2*

3: *S. coelicolor Δ1742/43*

4: *S. coelicolor Δ1742/45*

Figure S4: Expression of *SCO1742* in the different strains by RT-PCR. Amplification by RT-PCR of a region of *SCO1742* gene in the wild type strain *S. coelicolor* M145 and the different mutants Δ*abrA1/A2*, Δ*1742/43*, and Δ*1742/45*.
