# Supplementary material for: Regulation of the AbrA1/A2 Two-Component System in Streptomyces coelicolor and the Potential of Its Deletion Strain as a Heterologous Host for Antibiotic Production
Source: PLoS One. 2014 Oct 10;9(10):e109844. doi: 10.1371/journal.pone.0109844 (PMC4193843; doi:10.1371/journal.pone.0109844)
Supplement: Table S1 — Primers used in this work. (PDF) [file pone.0109844.s005.pdf]

Table S1 – Primers used in this work

Primers	Sequence (5´-3´)	Function
AY-006P	GAGCCCCCGCACACCGCGGCCCACGGTGTGC GGGGGCTCATGTAGGCTGGAGCTGCTTG	Reverse oligonucleotide for amplifying the mutagenesis cassette for *1742-45* genes. PAGE-purified.
AY-033	TTTTTTGAATTCCTCACGTCCTCCTGTGGGGGG	Forward oligonucleotide for amplifying the *abrA* promotor. The sequence recognized by EcoRI is underlined
AY-034	TTTTTTCATATGTCCGAAGCCTCCGGTGTGCC	Reverse oligonucleotide for amplifying the *abrA* promotor. The sequence recognized by NdeI is underlined
AY-035	TTTTTCATATGAACGACCTCTTCGGGCGGG	Forward oligonucleotide for cloning *abrA1*. The sequence recognized by NdeI is underlined
AY-036	TTTTTCTCGAGTCAGCCCGTGCCGCCGGGG	Reverse oligonucleotide for cloning *abrA2*. The sequence recognized by XhoI is underlined
AY-043	GGCACACCGGAGGCTTCGGACATGATTCCGGG GATCCGTCGACC	Forward oligonucleotide for amplifying the mutagenesis cassette for 1742/43 and 1742-45 genes. First PCR
AY-044	CCCGCCCGAAGAGGTCGTTCACTGTAGGCTGG AGCTGCTTC	Reverse oligonucleotide for amplifying the mutagenesis cassette for 1742/43 genes. First PCR
AY-045	TGGGTGGAGGACGGGGAGATCGGCACACCGG AGGCTTCGGAC	Forward oligonucleotide for amplifying mutagenesis cassette for 1742/43 and 1742-45 genes. Second PCR
AY-046	AACAGGTCGTACGCCGCCCGGGCCCGCCCGA AGAGGTCGTTCAC	Reverse oligonucleotide for amplifying the mutagenesis cassette for 1742/43 genes. Second PCR
AY-047	TTTTTTCATATGACCATCCGCCTGCTGATCG	Forward oligonucleotide for cloning *abrA2*. The sequence recognized by NdeI is underlined.
AY-048	TTTTTTCTCGAGGCCCGTGCCGCCGGGGAGG	Reverse oligonucleotide for cloning *abrA2*. The sequence recognized by XhoI is underlined.
AY-061	GGGCTCGCCGCGGCGCTCC	Forward oligonucleotide for RT-PCR of *SCO1742*.
AY-062	AACCGGCCTCCGTCGTCGGTC	Reverse oligonucleotide for RT-PCR of *SCO1742*.
AY-102	TTTTTTCTCGAGGATCGACGGCGACAGCAGGG	Reverse oligonucleotide for cloning N-terminal *abrA2*. The sequence recognized by XhoI is underlined.

AY-103	GGTCCTGATG**G**C**C**ATCCGCATGC	Forward oligonucleotide for the point mutation AbrA2_D55A (bold).
AY-104	GCATGCGGATGGCCATCAGGACC	Reverse oligonucleotide for the point mutationAbrA2_D55A.
SRG-045	CCGGTACGGATCAGTTCCTGGTCGTCGACGATCAGCAGGCGGATGGTCATTGTAGGCTGGAGCTGCTTC	Reverse oligonucleotide for amplifying the mutagenesis cassette for 1744.
SRG-046	TCGTGGTGACCGCCCGCCTGCCCGTCGAGGACGCCGGGGACGCGGCATGAGTGATTCCGGGGATCCGTCGACC	Forward oligonucleotide for amplifying the mutagenesis cassette for 1745.
SRG-047	TTTTTTCATATGAACGACCTCTTCGGGCGGG	Forward oligonucleotide for cloning 1744 (abrA1) with *xysAp*.
SRG-050	GGTCCTGATGGAAATCCGCATGC	Forward oligonucleotide for the point mutation AbrA2_D55E
SRG-051	GCATGCGGATTTCCATCAGGACC	Reverse oligonucleotide for the point mutation AbrA2_D55E.
SRG-058	GGCAGCGCGACGAGCCCCCGCACACCGCGGCCACGGTGTGCGGGGGCTCATGTAGGCTGGAGCTGCTTC	Reverse oligonucleotide for amplifying the mutagenesis cassette for 1745.
SRG-059	CGCGGTCACGCTGGTCGCGGTCGTGGTCGAGCGACGGCGCGATCTGGCGTGATTCCGGGGATCCGTCGACC	Forward oligonucleotide for amplifying the mutagenesis cassette for 1744.
SRG-060	TTTTTT<u>CATATG</u>ACCATCCGCCTGCTGATCGTCGACGCCCAGGAACTG	Forward oligonucleotide for the point mutation AbrA2_D10A.The sequence recognized by NdeI is underlined
SAM-001	TTTTTT<u>CTCGAG</u>TCATGCCGCGTCCCCGGCGTCC	Reverse oligonucleotide for cloning *abrA1.* The sequence recognized by XhoI is underlined
