# Supplementary material for: Regulation of the AbrA1/A2 Two-Component System in Streptomyces coelicolor and the Potential of Its Deletion Strain as a Heterologous Host for Antibiotic Production
Source: PLoS One. 2014 Oct 10;9(10):e109844. doi: 10.1371/journal.pone.0109844 (PMC4193843; doi:10.1371/journal.pone.0109844)
Supplement: Table S2 — Plasmids and cosmids used in this work. (PDF) [file pone.0109844.s006.pdf]

Table S2 - Plasmids and cosmids used in this work

Vector	Characteristics	Reference
SCI11	Supercos-1 derivative: *bla, neo*. Contains genes *SCO1744/45*	[1]
ΔSCI11-2	SCI11 *ΔSCO1744::aac(3)IV*	This work
ΔSCI11-3	SCI11 *ΔSCO1745::aac(3)IV*	This work
ΔSCI11-4	SCI11 *ΔSCO172-45::aac(3)IV*	This work
ΔSCI11-5	SCI11 *ΔSCO1742/43::aac(3)IV*	This work
pKC505	Shuttle cosmid vector *E. coli-Streptomyces*. Apramycin resistance	[2]
CosAB4	pKC505 derivative replicative cosmid containing the cluster of oviedomycin from *S. antibioticus*	[3]
pKC505ie	pKC505 shuttle cosmid integrative vector with PhiC31 integration site. Apramycin and erythromycin resistance	Lombo et al (unpublished results)
CosAB4ie	pKC505ie derivative integrative cosmid containing the cluster of oviedomycin from *S. antibioticus*	Lombo et al (unpublished results)
pNX4	pN702GEM3 derivative containing *xysA*Δ under *xysAp*	[4]
pNA4	pNX4 derivative containing *xysA* under *abrAp*	This work
pHabrA	Integrative plasmid containing *abrA1/A2*under*abrAp* with hygromycin resistance	[5]
pHabrA1	pHabrA derivative containing *abrA1*under *abrAp*	Thiswork
pHabrA2	pHabrA derivative containing *abrA2*under *abrAp*	Thiswork
pXHis1	*E. coli* plasmid ampicillin resistance containing *xysA*Δ under *xysAp*	[6]
pXabrA2His	pXHis1 derivative containing *abrA2* under the *xysA* promoter	This work
pIJ702	*Streptomyces* muticopy plasmid thiostreptone resistance	[7]
pTXabrA2	pIJ702 derivative containing *abrA2* under the *xysA* promoter	This work
pTXabrA2-DA	pTXabrA2 derivative expressing the AbrA2-D55A mutation	This work
pTXabrA2-DE	pTXabrA2 derivative expressing the AbrA2-D55E mutation	This work
pTXabrA2-DADE	pTXabrA2 derivativeexpressing AbrA2-D10A/D55E mutation	This work

pET22b	*E. coli* expression vector. Ampicillin resistance.	Novagen
pET22abrA2	pET22b derivative containing *abrA2* with a His6 tag at the carboxy terminal.	This work
pETabrA2$_N$	pET22b derivative containing a truncated *abrA2* with a His6 tag at the carboxy terminal. Corresponds to the first 141 AAs	This work

References

1. Redenbach M, Kieser HM, Denapaite D, Eichner A, Cullum J, et al. (1996) A set of ordered cosmids and a detailed genetic and physical map for the 8 Mb *Streptomyces coelicolor* A3(2) chromosome. Molecular Microbiology 21: 77-96.

2. Richardson MA, Kuhstoss S, Solenberg P, Schaus NA, Rao RN (1987) A new shuttle cosmid vector, pKC505, for streptomycetes: its use in the cloning of three different spiramycin-resistance genes from a Streptomyces ambofaciens library. Gene 61: 231-241.

3. Mendez C, Kunzel E, Lipata F, Lombo F, Cotham W, et al. (2002) Oviedomycin, an unusual angucyclinone encoded by genes of the oleandomycin-producer *Streptomyces antibioticus* ATCC11891. Journal of natural products 65: 779-782.

4. Rodríguez S, Santamaría RI, Fernández-Ábalos JM, Díaz M (2005) Identification of the sequences involved in the glucose-repressed transcription of the *Streptomyces halstedii* JM8 *xysA* promoter. Gene 351: 1-9.

5. Yepes A, Rico S, Rodriguez-Garcia A, Santamaria RI, Diaz M (2011) Novel two-component systems implied in antibiotic production in *Streptomyces coelicolor*. PLoS One 6: e19980.

6. Adham SA, Honrubia P, Díaz M, Fernández-Ábalos JM, Santamaría RI, et al. (2001) Expression of the genes coding for the xylanase Xys1 and the cellulase Cel1

from the straw-decomposing *Streptomyces halstedii* JM8 cloned into the amino-acid producer *Brevibacterium lactofermentum* ATCC13869. Arch Microbiol 177: 91-97.

7. Katz E, Thompson CJ, Hopwood DA (1983) Cloning and expression of the tyrosinase gene from *Streptomyces antibioticus* in *Streptomyces lividans*. Journal of General Microbiology 129: 2703-2714.
